# A ‘modified’ keystone flap in a single–surgeon case series: A new design in 32 patients

**DOI:** 10.4102/jcmsa.v3i1.189

**Published:** 2025-05-23

**Authors:** Samuel J. Isaacs, Saleigh Adams

**Affiliations:** 1 Department of Surgery, Faculty of Plastic Surgery, University of Cape Town, Cape Town, South Africa

**Keywords:** flap, plastic surgery, reconstruction, keystone flap, case series, modification, keystone perforator flap

## Abstract

**Introduction:**

The keystone island flap was described in 2003 by Dr Felix Behan. The design is a curvilinear, trapezoidal random perforator island flap with 4 classic subtypes (I–IV). It was named ‘keystone’ as it is identical in shape to the central pillar (keystone) used in architecture. It has shown its versatility in the closure of complex skin defects and wounds as an alternative to skin grafts and other reconstruction options (pedicled, transposition or rotation-advancement flaps). This case series reviews the practical design, use and applications of a ‘modified flap’. The key modification is the use of only lateral limb fasciotomy incisions to the keystone flap.

**Patients’ presentation:**

A retrospective single–surgeon case series (32 patients), each of whom underwent the ‘modified’ technique. Data were obtained from folder review on consenting adults who underwent surgery (01 January 2018 to 01 January 2022). Permission was obtained telephonically, and data were captured in a standardised sheet and electronically. Approval was granted by the ethical review board.

**Management and outcome:**

Defects (4 cm^2^ – 72 cm^2^) were reconstructed in adults (21–94 years). The most common site was the lower limb (23/32). There were two minor cases of epidermolysis and no flap loss (100% success rate).

**Conclusion:**

The ‘modified’ keystone flap is versatile and reliable with applications in multiple sites as a reconstructive tool.

**Contribution:**

The authors propose a modification to the existing system to include a Type IIC with the ‘modified’ design.

## Introduction

In his seminal article in 2003, Behan describes the logic of the naming of the flap and the techniques of flap design and raising.^1^ This article introduces the concept of the four major subtypes of the keystone flap and is not a surgical case series but an introduction to the technique and design of the flap with illustrative isolated and single cases.^1^ It provides detailed and clear guidelines about the situations in which one raises the various types of flaps and demonstrates in which situations each subtype of flap is used. Conceptually, the flap subtypes are defined as subtypes I–IV.^1^

Type I is employed for very small defects measuring 2 cm or less in diameter. It comprises a trapezoidal, curvilinear flap with skin incisions only where the defect is closed without the use of a skin graft, as there is sufficient laxity to close the wound primarily.^1^ Key points to the Type I flap are the angle of the lateral limbs, which are placed at 90° and the width of the defect to flap size, which is 1:1. The angle of the lateral limbs is designed as the movement of the flap and is based on the V-Y closure or movement of these limbs. Illustrations demonstrating the four traditional classification types of keystone island perforator flaps, as originally described by Behan are discussed next together with brief descriptions of the subtypes.^1^

Type IIA is for defects that measure more than 2 cm. The resulting tissue recruited to close the flap requires not only skin incisions but also fascial incisions into the base of the flap and the lateral limbs of the flap.^1^ This has the potential to disrupt or compromise the blood supply of the flap because the blood supply to the flap is from the fascial base underneath the skin. In addition, there is a random–pattern dermal component to the blood supply of the flap.

The Type IIB keystone flap is identical in design to the Type IIA keystone flap but it requires a skin graft to close the donor site or adjacent site from where the tissue was recruited.^1^ The lateral sides of the flap and the larger curve of the flap are incised.

The Type III keystone flap comprises two identical, opposing keystone flaps that are used to create a double keystone flap.^1^

The Type IV (rotational keystone) is a keystone flap design that is ideally suited as an alternative to free flap cover for compound tissue loss and exposed bone on the lower limb. The keystone flap is raised with undermining of 50% of the flap subfascial. Blood to this flap is supplied by the perforators from the adjoining skin.^1^

**FIGURE 1:**
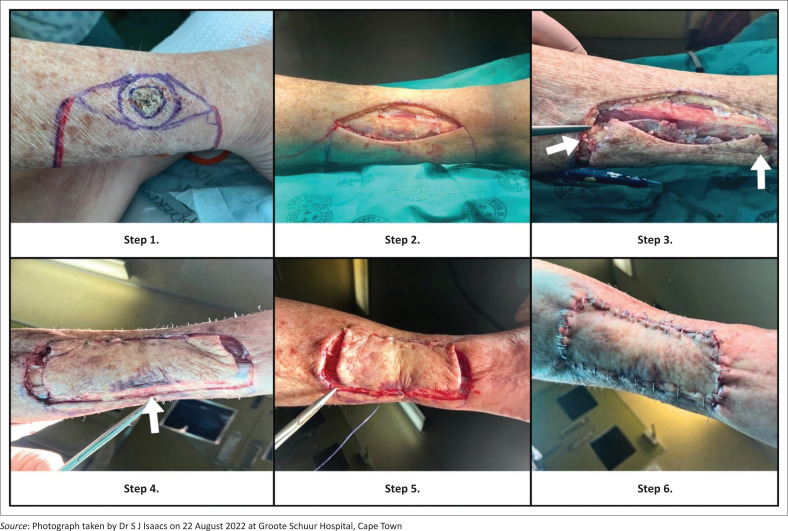
Surgical technique of the modified flap design.

The senior author proposes a modification to the existing technique as illustrated in Figure 1 – a Type IIC.

Behan followed his seminal 2003 article with a second retrospective surgical case series titled *Keystone Island Flap: An Alternative Reconstructive Option to Free Flaps in Irradiated Tissue*.^2^ This article was a single–surgeon retrospective case series of seven patients who underwent head and neck reconstruction for skin cancer.^2^ This small series showed that closure of the created defect post excision was effective and had low patient morbidity in an elderly patient population with comorbidities.^2^ This was often in patients who had received or who were to receive postoperative radiotherapy.^2^

Since 2003, there have been a number of key articles showing the efficacy and versatility of the keystone flap as originally described by Behan.^1,3,4,5,6,7,8,9^ A literature review of these article shows the versatility and robustness of the keystone flap and its versatility in closing complex defects, even avoiding complex head and neck microsurgery reconstruction.^2,3,4,5,6,7,8,9^ This is of particular relevance on the African continent where there is a paucity of resources and limited access to complex microsurgical reconstruction.

The versatility by location has been highlighted in publications by Mohan et al., which focus on the usefulness of the keystone flap in upper and lower limb reconstruction.^9^ Other modifications include the increase in the ratio of defect to flap size from 1:1 to 1:3 in the lower limb with minimal morbidity or complications. The latter was described in the 2015 article by Rao and Janna.^10^

Despite the usefulness and clinical evidence of the robustness of the keystone flap, there is controversy relating to the vascularity and microcirculatory basis of the flap. The only anatomically based study on the keystone flap was conducted by Lo et al., and it shows that the post-flap inset changes are not only related to improvements in the perforator base of the flap and the cutaneous but also to neurocutaneous changes.^11^ Further basic science research is needed to understand the physiological basis of this flap.^11^ To date, there are no updated scientific or experimental studies relating to the vascularity of the keystone flap. Despite the paucity of evidence, the value and robustness of the keystone flap are evident and are based on the success of the flap and its acceptance as a workhorse option for reconstruction.^11^

There have been several modifications to the basic design of the keystone flap. However, none of these designs uses a lateral fasciotomy incision.^11,12,13,14,15,16,17,18,19,20^

None of the studies in the literature describes the technique designed by the senior author of this article. The technique of this modified flap is to use lateral fasciotomy incisions to the sides of the trapezoid shape only. This technique theoretically does not disturb the blood supply of the larger curvature of the flap or the base of the flap, which represents a larger surface area.

## Surgical technique of modified flap

The surgical technique for the modified flap design is illustrated here with surgical sequencing:

**Step 1:** Flap design and marking. Marking of the lesion – in this case, confirmed basal cell carcinoma (BCC) – with an appropriate surgical margin

**Step 2:** Excision of the BCC, with the final defect requiring modified keystone reconstruction

**Step 3:** Lateral fasciotomy incisions down to and through deep fascia

**Step 4:** Preservation of deep fascia at base of ‘modified’ keystone flap

**Step 5:** Inset of ‘modified’ keystone flap with deep dermal sutures

**Step 6:** Final surgical result with inset of ‘modified’ keystone flap

Hence, the senior author proposes a change to the existing classification system to include the Type IIC ‘modified’ flap.

## Aims and objectives

The aims of this retrospective case series of 32 flaps in 32 patients were as follows:

- To review the clinical outcome defined by flap survival in the newly ‘modified’ keystone flap design and to ascertain the defect sites and the sizes that were closed by this modified surgical technique.
- The primary objective was to ascertain if this modification of the keystone flap was a safe and reliable soft tissue option as measured clinically by flap survival in a single–surgeon case series.
- The secondary objective was to ascertain the sizes of the defect sites that were successfully closed in this surgical case series using the modified flap.

## Patient presentation

This was a single–surgeon case series of 32 flaps in 32 patients. The study design was a retrospective surgical case series with data collected via a folder review.

Patients were from a tertiary hospital system in which patients were referred to a plastic and reconstructive surgery unit for treatment. The primary setting was a tertiary academic Hospital in the Western Cape. Data were obtained from a folder review on consenting adults who underwent surgery for various pathologies from 01 January 2018 to 01 January 2022.

Ethics and institutional approval to conduct the study was received with a relevant study number.

Patient permission was obtained, and the data were captured in a standardised sheet and electronically recorded. The data were then collated into descriptive datasets by age, demographics, anatomical location of defect, pathology and complications.

There were no barriers to the data collection, as the data had been recorded in the patients’ clinical folders by the senior author.

Patients were initially followed up by the senior surgeon one day post-surgery until 3 months. The patients were reviewed at regular intervals for signs of infection and flap survival and initially, vascularity. Skin staples and sutures were removed after 10 days as a standard.

### Inclusion criteria

All consenting adult patients (older than 18 years of age) who had undergone ‘modified’ keystone flap reconstruction for surgically indicated pathologies from 01 January 2018 to 31 December 2020 were eligible.

### Exclusion criteria

Patients who were less than 18 years of age and patients who had undergone non-modified keystone flap reconstructions (the classical I–IV types as described by Behan^1^) were rejected. In addition, patients who had undergone keystone flap surgery prior to 01 January 2018 were ineligible as the non-modified reconstructive type of flap was used.

### Statistical analysis

The data were analysed using mean, standard deviation or median where appropriate. If required, for paired comparison, the Student’s *t*–test and the Fisher’s exact test are used to analyse statistically the relationship between the groups. A *p*–value of < 0.05 is regarded as significant. However, the Student’s *t*-test and the Fisher’s exact test were not required in this study, as this single–surgeon case series was a descriptive study and there were no statistical comparisons between groups.

### Ethical considerations

Ethical clearance to conduct this study was obtained from the University of Cape Town Faculty of Health Sciences Human Research Ethics Committee. (No. 636/2022).

## Management and outcome

The study sample consisted of male participants (*n* = 24) and female participants (*n* = 8). The age range of the study population was 21–94 years. The average age of the study population was 67 years.

The most common comorbidity was hypertension, followed by hypercholesterolaemia. In addition, 7 of the 32 participants were non-insulin-requiring patients with diabetes. The average defect size was 19 cm^2^, with a range of 4 cm^2^ – 72 cm^2^.

The most common site for surgery was the lower leg. There were 23 cases requiring surgery on the lower leg. There was one case involving the neck, two involving the trunk, one involving the axilla, two involving the upper arm, one involving the lower arm and two cases involving the upper leg.

The most common indication was post-ablative skin cancer reconstruction for BCC (16 of the 32 patients). Other indications were squamous cell carcinoma (7 of the 32 patients), post-sepsis reconstruction (5 of the 32 patients) and post-melanoma excision (3 of the 32). Only one patient received a reconstruction for acne keloidalis nuchae excision of the occipital scalp or neck area.

The Student’s *t*-test and Fisher’s exact test were not required in this study, as this single–surgeon case series was a descriptive study and there were no statistical comparisons between groups.

Regarding complications of the total study population, only 2 of the 32 cases developed superficial epidermolysis of the skin flap. Hence, there were two partial flap losses. All the remaining 30 of the 32 flaps survived and were successful. The management of these two patients was conservative with daily dressings, and the healing was uneventful. The flap survival was, therefore, 100% in this case series of patients.

## Discussion

The original publication of the keystone flap was from Behan in 2003.^1^ Since its original description and publication, the keystone flap has joined the armamentarium of the plastic surgeon to reconstruct both simple and complex wounds.^1,2,3,4,5,9,12,13,14,15,16,17,18,19^ It is a valuable and viable option for a number of reconstructive surgery solutions, as it does not require specialised microsurgery instrumentation or infrastructure.^2^ This is very relevant in resource-limited environments such as Africa.

In this surgical case series, the ‘modified’ keystone flap and reconstruction were successful in a wide age range of patients (21–94 years old) with a wide range of surgical indications. In this series, the size of the defects that were closed with this technique ranged from 4 cm^2^ to 72 cm^2^. This study demonstrated the robustness and versatility of the ‘modified’ keystone flap in the reconstruction of a range of defects in various anatomical sites with different pathologies. This was illustrated by flap survival of the 32 flaps, with only minor complications in 2 of the 32 patients. The overall survival rate of the flap was 100%. Superficial epidermolysis is a recognised complication in flap surgery, and both these patients were managed conservatively without loss of the flap or the need for further surgery.

A summary of the study population is presented in Table 1.

**TABLE 1:** Patient, side, surgical site and size in cm.

Patient no.	Side	Surgical site	Size of defect (cm)
1.	Right	Upper leg	12 × 6
2.	Left	Lower leg	7 × 3
3.	Left	Upper arm	10 × 5
4.	Left	Lower leg	2 × 3
5.	Right	Lower leg	2.3 × 3.3
6.	N/A	Trunk	2 × 3
7.	Left	Lower leg	2 × 2
8.	Right	Axilla	6 × 3
9.	Right	Lower leg	9 × 4
10.	Right	Lower leg	6 × 3
11.	N/A	Neck	2 × 2
12.	Left	Lower leg	2 × 2
13.	Left	Lower leg	2 × 4
14.	Left	Lower arm	2 × 2
15.	Right	Lower leg	11 × 4
16.	Right	Upper arm	10 × 3
17.	Right	Lower leg	6 × 2
18.	Left	Lower leg	7 × 3 × 5
19.	Left	Lower leg	6 × 4
20.	Left	Lower leg	3 × 2.5
21.	Right	Lower leg	10 × 5
22.	Right	Lower leg	4 × 3
23.	N/A	Trunk	6 × 3.5
24.	Left	Lower leg	8 × 3
25.	Right	Lower leg	4 × 4
26	Right	Lower leg	10 × 5.5
27.	Right	Lower leg	2 × 2
28.	Left	Lower leg	2 × 3
29.	Right	Lower leg	4 × 4
30.	Right	Lower leg	2 × 2
31.	Left	Lower leg	2 × 2
32.	Left	Lower leg	2 × 2.5

A number of modifications have been described in the literature regarding the keystone flap.^9,10,11,12,13,16,17,18,19^ These modifications to the flap design have been of a geometric nature to decrease the need for a skin graft and to improve the advancement of the flap in specific anatomical locations.^9,10,11,12,13,16,17,18,19^ The modification described in this study is, however, unique in the lateral fasciotomy, as illustrated in the surgical technique. There are no reports of modified techniques as described and pioneered by the senior author.

Lateral fasciotomy incisions aid in the closure of the wound by assisting flap advancement and protecting the blood supply of the flap by maintaining the fascial base. In addition, the technique is easy to perform and it does not involve complex surgery. These are the primary advantages of this modified technique over the other described techniques. Moreover, the success rate of this modification is comparable with other described modifications.^11,12,13,14,15,16^

The technique of this modified flap uses skin and lateral fasciotomy incisions only to the lateral sides of the trapezoidal keystone flap and a skin–only incision to the outer curvilinear base. The lateral fasciotomy incisions aid in the closure of the wound by not only separating the epidermal and dermal connections but also by releasing the lateral fascial attachments.^1,2,3,4^ In a conventional advancement flap, the subcutaneous skin and the fascia are released, creating an islanded flap that can be advanced.^4,5,6,^ However, in this modified technique, the advancement at the V-Y closure site at these lateral fasciotomy sites is enhanced and the outer curvilinear fascial site requires a skin-only incision, thus preserving perfusion at this site. This is in contrast with a conventional advancement flap in which the skin or the skin and fascia are islanded and advanced.^4,5^ In this modification, there is maximal use of the lateral V-Y advancement and closure without the need for using Burrow’s triangles or additional techniques as the fascia folds on itself, which is something that soft tissue will not do in a conventional flap.^1,2,3,4^

Limitations of the study include its retrospective nature, the small sample size and all the limitations that are related to these, including incomplete notes, missing variables and incomplete variables. An example is that the smoking status of the patient was not observed and was thus excluded from the data collection. However, successful reconstruction was performed in several patients with comorbidities, attesting to the robustness of the design.

Controversy regarding the keystone flap in general centres on the vascular basis for the flap and this was highlighted by Douglas et al.^20,21^ The lack of experimental and vascular studies of the keystone flap challenged the scientific and experimental validity of the flap. The question of the vascular basis of the keystone flap was addressed by Lo et al.^11^ This was underpinned by an improvement in the understanding of perforasome blood supply to the flap and the evolving concepts, as highlighted by Saint-Cyr,^6^ Saint-Cyr et al.^7^ and Mohan et al.^9^ The anatomical basis for the keystone was, however, upheld, and this is evidenced by the overwhelming clinical success of the flap and its robustness in plastic and reconstructive surgery.^11^

Questions that remain unanswered pertain to the actual vascularity changes of the keystone flap.^11,15^ There are limited experimental and vascular studies of the keystone flap despite its overwhelming clinical success, and further experimental studies related to this flap are needed.^11,15^

**FIGURE 2:**
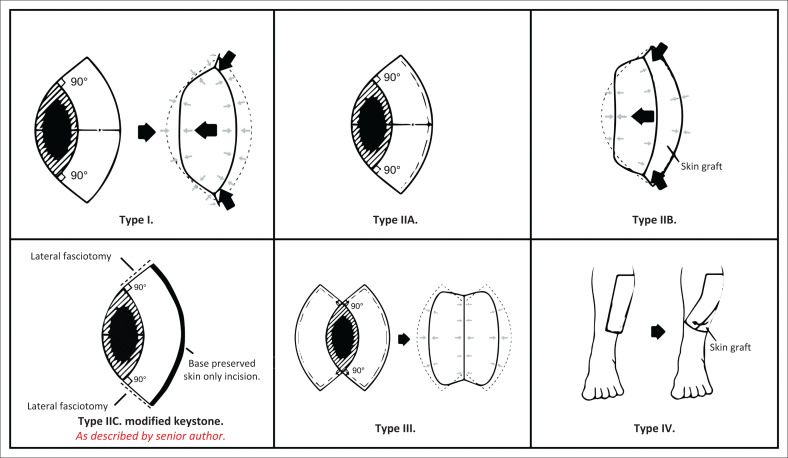
Proposed new classification system to include Type IIC and the classical types I–IV of Behan.^1^

Because of the clinical outcomes and published literature regarding the keystone flap, it remains an invaluable tool for reconstruction by plastic surgeons. This study highlights a simple, safe and reproducible modification to the flap design that is practical and original. Based on this study, the recommendation of the senior author is to modify the original flap classification described by Behan^1^ to include a type with only lateral fasciotomy incisions, thus sparing the base of the keystone design. This would be a Type IIC. This is illustrated in Figure 2.

## Conclusion

This study shows the robustness and versatility of the ‘modified’ keystone flap in a retrospective surgical series of 32 ‘modified’ keystone flap reconstructions. This new technique and flap design is robust, original and practical.

The senior author recommends a change in the traditional and original keystone flap classification to include the Type IIC, which involves only lateral fasciotomy incisions. This aids in the advancement of the flap towards closure of the defect and protects the vascularity by maintaining the fascial base of the flap.
